# Intoxication by Intraperitoneal Injection or Oral Gavage Equally Potentiates Postburn Organ Damage and Inflammation

**DOI:** 10.1155/2013/971481

**Published:** 2013-11-28

**Authors:** Michael M. Chen, Jessica L. Palmer, Jill A. Ippolito, Brenda J. Curtis, Mashkoor A. Choudhry, Elizabeth J. Kovacs

**Affiliations:** ^1^Burn and Shock Trauma Research Institute, Loyola University Chicago, Health Sciences Campus, Maywood, IL 60153, USA; ^2^Alcohol Research Program, Loyola University Chicago, Health Sciences Campus, Maywood, IL 60153, USA; ^3^Loyola University Chicago, Health Sciences Campus, 2160 South First Avenue, Maywood, IL 60153, USA; ^4^Department of Surgery, Loyola University Chicago, Health Sciences Campus, Maywood, IL 60153, USA

## Abstract

The increasing prevalence of binge drinking and its association with trauma necessitate accurate animal models to examine the impact of intoxication on the response and outcome to injuries such as burn. While much research has focused on the effect of alcohol dose and duration on the subsequent inflammatory parameters following burn, little evidence exists on the effect of the route of alcohol administration. We examined the degree to which intoxication before burn injury causes systemic inflammation when ethanol is given by intraperitoneal (i.p.) injection or oral gavage. We found that intoxication potentiates postburn damage in the ileum, liver, and lungs of mice to an equivalent extent when either ethanol administration route is used. We also found a similar hematologic response and levels of circulating interleukin-6 (IL-6) when either ethanol paradigm achieved intoxication before burn. Furthermore, both i.p. and gavage resulted in similar blood alcohol concentrations at all time points tested. Overall, our data show an equal inflammatory response to burn injury when intoxication is achieved by either i.p. injection or oral gavage, suggesting that findings from studies using either ethanol paradigm are directly comparable.

## 1. Introduction

Ethanol is the most commonly abused substance in the United States and the third leading cause of preventable death [1], many of which are associated with unintentional injuries [2]. Binge drinking, defined as reaching a blood alcohol content of 0.08 [3], in particular, is an increasingly prevalent form of intoxication [4] and the characteristic drinking pattern of trauma patients [5]. As a central nervous system depressant, alcohol likely plays a causative role in many accidents but the diverse cellular effects of alcohol and its metabolites can also negatively alter the physiologic response to injury [6]. As a small neutral compound capable of freely traversing lipid membranes, alcohol can influence nearly every cell in the body with effects dependent on the amount and duration of exposure [7]. Even a single dose of alcohol in animals has been shown to worsen systemic inflammation after injuries, such as burns [8, 9]. Burns are a devastating injury with a complex natural history and high association with alcohol [10]. Nearly half of adult burn patients have a positive blood alcohol concentration (BAC) at the time of admission and this predisposes them to worsened clinical outcomes compared to patients with similar injuries not under the influence [11]. Specifically intoxicated patients were found to be twice as likely to acquire an infection, required more surgical procedures, had longer durations of stay in the intensive care unit, and generated more cost than their nonintoxicated counterparts [12]. Interestingly, these patients are not typically chronic alcoholics but are considered binge drinkers [13], consistent with the majority of alcohol consumption in the US [4]. With nearly 450,000 burns requiring medical attention each year in the American healthcare system [14], alcohol greatly contributes to the socioeconomic burden of this destructive injury as both a causative agent and complicating factor in recovery. Despite the high prevalence and established consequences of binge intoxication at the time of burn injury, there are currently few differences in the treatment and management of burn patients with and without prior alcohol exposure. This may be due in part to the aforementioned dynamic natural history of burns as well as the complex and duration dependent effects of alcohol. In order to develop much needed targeted therapies, the effects of intoxication on the physiologic response to burn injury need to be studied and manipulated under controlled conditions. To this end, mouse models of binge ethanol exposure and burn have been in use for nearly 20 years and yielded insightful information into the mechanisms by which ethanol exacerbates the response to burn. Of note is the finding that ethanol can potentiate burn-induced damage in the intestine [15, 16], liver [17, 18], and lungs [19, 20] with increased serum interleukin-6 playing an important role in inflammation of these organs [21, 22]. The majority of these studies administer ethanol by gavage or i.p. injection to reach a desired BAC in mice. While presumably the presence and level of intoxication are the most important factors in these models, no one to date has investigated the impact of the route of ethanol administration in the context of burn. It is important to establish if results from historical experiments using i.p. injection and gavage are directly comparable as well as be aware of any unintentional confounding factors in future studies. Herein we examine the effects of ethanol, given by gavage or i.p. injection, on postburn inflammation and damage in the intestines, liver, and lungs of mice.

## 2. Materials and Methods

### 2.1. Mice

Male wild-type (C57BL/6) mice were purchased from Jackson Laboratories (Bar Harbor, ME) and sacrificed at 8–10 weeks old. Mice were housed in sterile microisolator cages under specific pathogen-free conditions in the Loyola University Medical Center Comparative Medicine facility. All experiments were conducted in accordance with the Institutional Animal Care and Use Committee.

### 2.2. Murine Model of Ethanol and Burn Injury

A murine model of a single binge ethanol intoxication and burn injury was employed using either i.p. injection or oral gavage as described previously [23, 24]. Briefly, i.p. mice were given a single i.p. dose of 150 *μ*L of 20% (v/v) ethanol solution (1.12 g/kg) or saline control. Gavaged mice were given a single dose of 300 *μ*L of 10% (v/v) ethanol solution (1.12 g/kg) or water control. The mice were then anesthetized (100 mg/kg ketamine and 10 mg/kg xylazine), their dorsum was shaved, and they were placed in a plastic template exposing 15% of the total body surface area and subjected to a scald injury in a 92–95°C water bath or a sham injury in room-temperature water. The scald injury results in an insensate, full-thickness burn [25]. The mice were then resuscitated with 1.0 mL saline and allowed to recover on warming pads. All experiments were performed between 8 and 9 am to avoid confounding factors related to circadian rhythms.

### 2.3. Blood Alcohol Concentration (BAC) 

Mice were sacrificed at 30 minutes, 1 hour, or 4 hours after a single dose of ethanol (1.12 g/kg) administered by either i.p. injection or gavage. Whole blood was collected via cardiac puncture, incubated at room temperature for 20 minutes and then centrifuged at 3000 rpm at 4°C for 20 minutes. Serum was isolated and BAC was measured using the GM7 Micro-Stat Analyzer (Analox, Lunenburg, MA).

### 2.4. Blood and Serum Measurements

At 24 hours after injury mice were euthanized, blood was collected via cardiac puncture, and an aliquot was placed into a microcapillary tube and read for a complete blood count with differential by Hemavet (Drew Scientific, Dallas, TX). The remaining blood was harvested for serum as described above and stored at −80°C. Serum aliquots were used to measure IL-6 by enzyme linked immunosorbent assay (ELISA) (BD Biosciences, Franklin Lakes, NJ) or liver transaminase levels using a DRI-CHEM 7000 (HESKA, Loveland, CO).

### 2.5. Histopathologic Examination of the Ileum and Liver

At 24 hours after injury mice were euthanized and the ileum, liver, and lungs were harvested. The ileum was fixed overnight in 10% formalin, embedded in paraffin, sectioned at 5 *μ*m, and stained with hematoxylin and eosin (H & E). The length of 5 individual villi in 5 fields of view (100x) was measured for a total of 25 measurements per animal. The average was considered representative of the villus length in the ileum and demonstrative images are presented herein. The whole liver was removed at the time of sacrifice, weighed, and normalized to total body weight.

### 2.6. Bacterial Translocation

Bacterial translocation was assessed as previously described [26]. Briefly, 3–5 mesenteric lymph nodes per mouse were removed, placed in cold RPMI, and kept on ice. Nodes were separated from connective tissue and homogenized with frosted glass slides. Homogenates were plated on tryptic soy agar and incubated at 37°C overnight.

### 2.7. Histopathologic Examination of the Lung

The upper right lobe of the lung was inflated with 10% formalin and fixed overnight as described previously [27], embedded in paraffin, sectioned at 5 *μ*m, and stained with hematoxylin and eosin (H & E). Photographs were taken in a blinded fashion of 10 high power fields (400x) per animal and analyzed using the Java-based imaging program ImageJ (National Institutes of Health, Bethesda, MD). The images were converted to binary to differentiate lung tissue from air space and then analyzed for the percent area covered by lung tissue in each field of view as described previously [21]. Neutrophils were counted in a blinded fashion in 10 high power fields (400x).

### 2.8. KC Analysis of Lung Homogenates

The right middle lung lobe was snap-frozen in liquid nitrogen. The tissues were then homogenized in 1 mL of BioPlex cell lysis buffer according to manufacturer's instructions (BioRad, Hercules, CA). The homogenates were filtered and analyzed for cytokine production using an ELISA for KC (BD Biosciences, Franklin Lakes, NJ). The results were normalized to total protein using the BioRad protein assay (BioRad, Hercules, CA).

### 2.9. Statistical Analysis

Statistical comparisons were made between i.p. and gavage animals in the sham vehicle, sham ethanol, burn vehicle, and burn ethanol treatment groups, resulting in 8 total groups analyzed. One-way analysis of variance was used to determine differences between treatment responses, and Tukey's post hoc test once significance was achieved (*P* < 0.05). Data are reported as mean values ± the standard error of the mean.

## 3. Results 

### 3.1. Blood Alcohol Concentration Is Equal after I.P. Injection or Gavage

**Figure 1:**
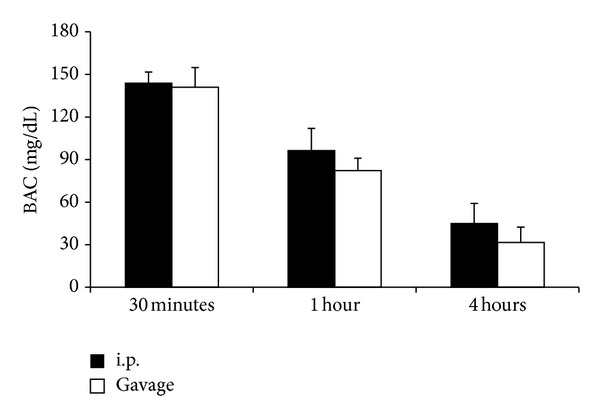
Blood alcohol concentration (BAC). Mice were administered 1.12 g/kg ethanol and the subsequent BAC measured at various time points. Data are presented at mean values ± SEM. *N* =  6–9 animals per group.

To determine if the route of ethanol administration impacted the kinetics of its absorption and clearance, the blood alcohol concentration (BAC) of mice was determined at 30 minutes, 1 hour, and 4 hours after a single dose of 1.12 g/kg ethanol by i.p. injection or gavage. Mice receiving ethanol by i.p. injection were found to have a BAC of 143 mg/dL at 30 minutes, which was reduced by 33% by 1 hour and 69% by 4 hours (Figure 1). Similarly, mice receiving ethanol by gavage demonstrated a BAC of 141 mg/dL at 30 minutes, which by 1 hour was decreased by 41% and by 77% at 4 hours (Figure 1). No significant difference between BAC in mice receiving ethanol via i.p. injection or gavage at each time point was found, suggesting that equivalent amounts of ethanol are absorbed into the bloodstream and are cleared at similar rates.

### 3.2. Intoxication by I.P. Injection or Gavage Increases Peripheral Blood Granulocytes after Burn

**Figure 2:**
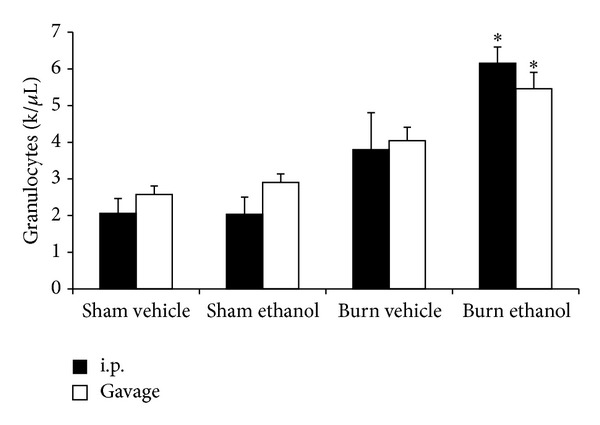
Circulating blood granulocytes 24 hours after injury. *P < 0.05 compared to Sham groups. Data are presented at mean values ± SEM. *N* =  3–6 animals per group.

To examine if administration route effected the hematologic response to intoxication and burn, the number of circulating granulocytes was enumerated by an automated counter after burn or sham injury when preceded by ethanol given by i.p. injection or gavage. In gavaged mice, there was a 2-fold increase (*P* < 0.05) in blood granulocytes in intoxicated burned mice relative to sham injured mice regardless of prior intoxication status (Figure 2). Likewise in mice given an i.p. ethanol injection before burn, a 3-fold increase (*P* < 0.05) in blood granulocytes was found when compared to sham injured mice with and without prior intoxication (Figure 2). No significant differences were found between i.p. injected and gavaged mice within treatment groups suggesting that both routes of ethanol administration induce an equal neutrophilic leukocytosis after burn injury.

### 3.3. Serum IL-6 Is Elevated When Intoxication Precedes Burn Injury regardless of Administration Route

**Figure 3:**
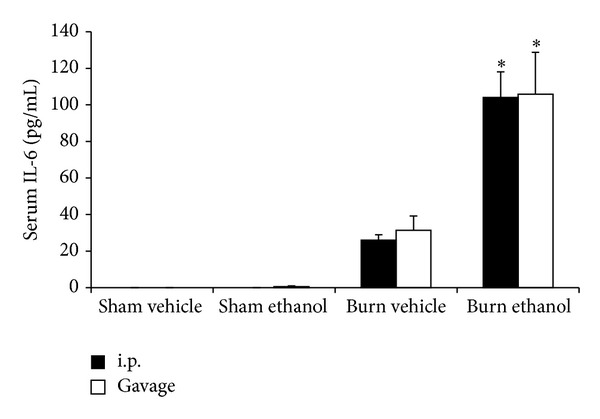
Serum IL-6 at 24 hours after injury. *P < 0.05 compared to Sham groups. Data are presented at mean values ± SEM. *N*= 4–8 animals per group.

Circulating IL-6 levels were quantified by ELISA in all treatment groups of i.p. injected and gavaged mice. Burn injury alone increased the amount of serum IL-6 by greater than 25-fold above sham injured animals in both i.p. and gavage mice (Figure 3). When intoxication preceded the burn, a further 3- to 4-fold increase (*P* < 0.05) above burn alone was observed, regardless of the route of ethanol administration (Figure 3). No significant differences were found between i.p. and gavage mice within treatment groups suggesting that intoxication before burn injury increases serum IL-6 irrespective of the ethanol paradigm used.

### 3.4. Villus Blunting Is Similar in I.P. and Gavage Intoxicated Mice after Burn

**Figure 4:**
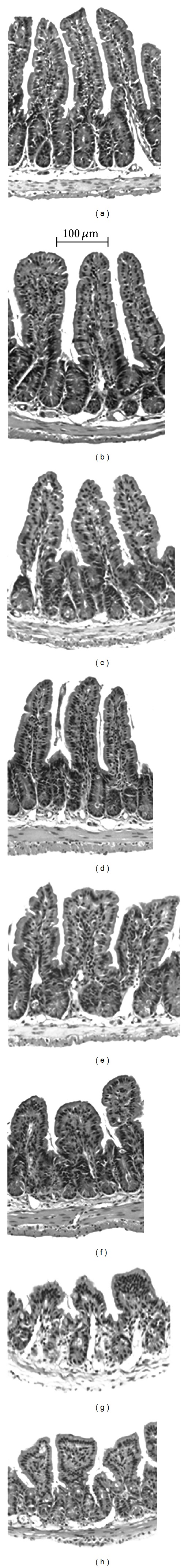
Histologic state of the ileum 24 hours after intoxication and burn injury. Sham injured mice receiving i.p. (a) and gavage (b) control, or i.p. (c) and gavage (d) ethanol have normal appearing villi. Burn injury alone receiving i.p. (e) and gavage (f) control demonstrate rounded and widened villi that are markedly blunted when combined with ethanol by i.p. injection (g) or gavage (h).

We previously reported that intoxication by i.p. injection furthers the diminution of ileal villi after burn [26]. Consistent with our earlier observations, at 24 hours after burn (Figures 4(e)-4(f)), villi in the ileum were shortened in comparison to sham injured animals regardless of intoxication status (Figures 4(a)–4(d)). Furthermore when mice were intoxicated by i.p. injection (Figure 4(g)) or gavage (Figure 4(h)), villus blunting was pronounced beyond burn alone.

**Figure 5:**
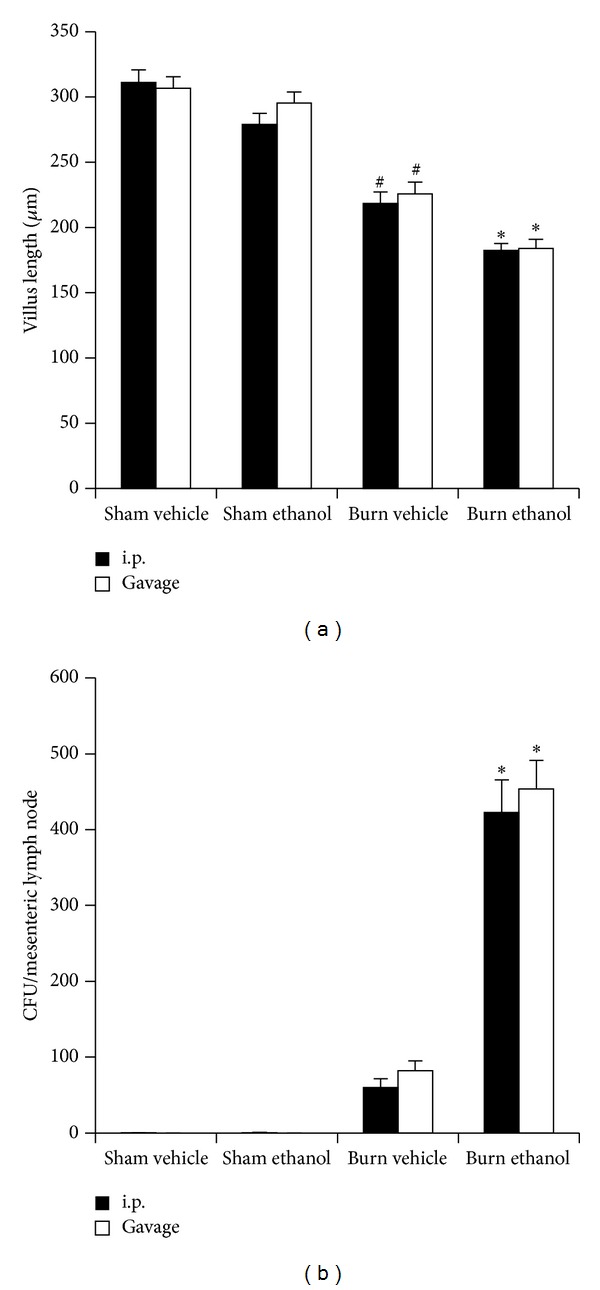
Villus length in the ileum (a) and bacterial load per mesenteric lymph node (b) 24 hours after injury. *P < 0.05 compared to Sham and Burn Vehicle groups. ^#^ *P* < 0.05 compared to Sham groups. Data are presented at mean values ± SEM. *n* = 4–6 animals per group.

The average villus length in the ileum of burn injured mice was blunted by greater than 20% (*P* < 0.05) compared to sham injured animals regardless of intoxication status or administration method (Figure 5(a)). Antecedent intoxication by i.p. injection or gavage caused a further ~20% reduction (*P* < 0.05) compared to burn alone (Figure 5(a)), demonstrating that this increased intestinal damage is present to a similar extent whether intoxication is achieved by i.p. injection or gavage. This villus blunting corresponded to an increase in bacterial translocation to the mesenteric lymph nodes where intoxication increased the number of colony forming units by >400-fold over sham animals and 5-fold over burn alone (Figure 5(b)). No significant differences between i.p. and gavage mice were found within treatment groups suggesting that both routes of ethanol administration effect intestinal damage after burn injury in a similar manner.

### 3.5. I.P. or Gavage Intoxication Equally Exacerbates Hepatic Damage after Burn

**Figure 6:**
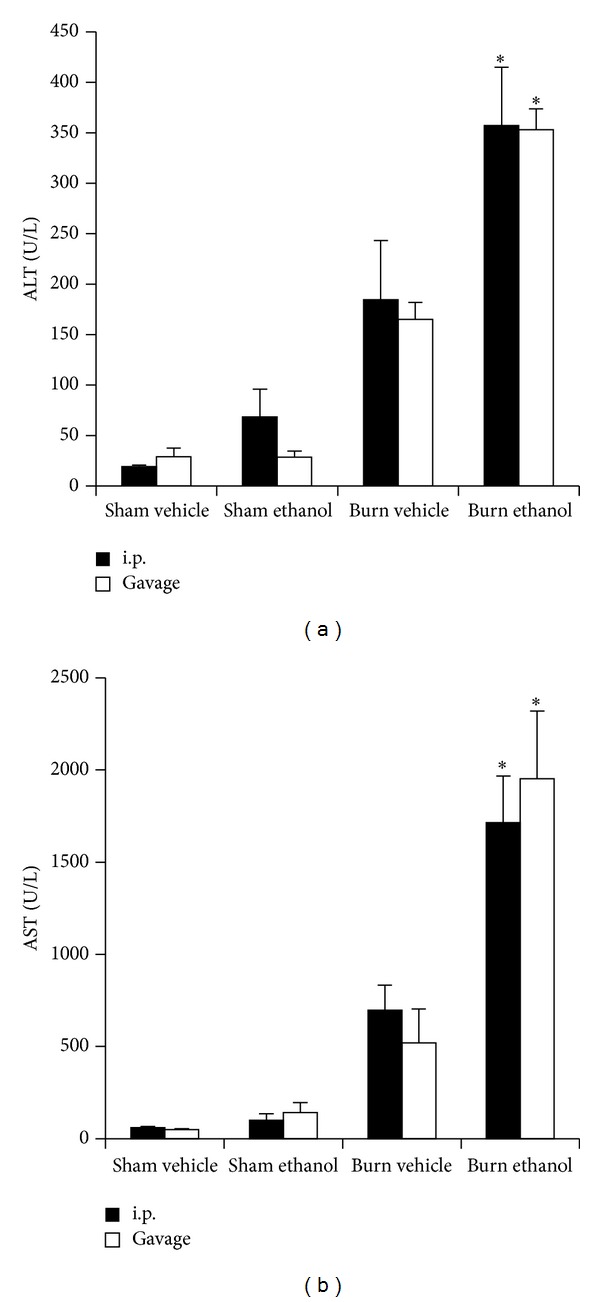
Serum alanine aminotransferase (ALT) (a) and serum aspartate aminotransferase (AST) (b) 24 hours after injury. *P < 0.05 compared to Sham and Burn Vehicle groups. Data are presented at mean values ± SEM. *N* =  3–5 animals per group.

Hepatic damage was measured by levels of serum alanine aminotransferase (ALT) and aspartate aminotransferase (AST) and by the liver weight to total body weight ratio. Burn alone increased serum ALT levels by greater than 6-fold compared to sham injured animals (Figure 6(a)). This increase was found irrespective of the presence or absence of ethanol or route of administration before sham injury. When i.p. or gavage mice were intoxicated at the time of burn, however, a greater than 12-fold elevation (*P* < 0.05) was observed over sham injured animals which corresponded to a ~2-fold increase (*P* < 0.05) above burn alone (Figure 6(a)). A similar pattern was found for serum AST with burn alone causing a greater than 5-fold increase relative to sham injured groups in both i.p. injected and gavaged mice with and without ethanol (Figure 6(b)). Intoxication in both i.p. and gavage mice at the time of burn increased serum AST an additional 2-fold (*P* < 0.05) which is a greater than 20-fold elevation (*P* < 0.05) over sham injured mice (Figure 6(b)).

**Figure 7:**
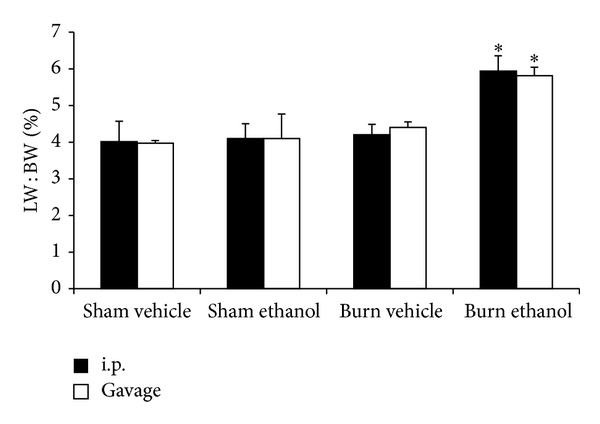
Liver weight (LW) to body weight (BW) ratio 24 hours after injury. *P < 0.05 compared to Sham and Burn Vehicle groups. Data are presented at mean values ± SEM. *N* = 4–6 animals per group.

Finally, the liver weight to total body weight ratio (LW : BW) was recorded as a measure of hepatic edema. No significant changes in LW:BW were found between Sham groups regardless of ethanol intoxication or administration route (Figure 7). Similarly, burn alone did not cause a significant change in LW : BW relative to sham injured mice. However when mice received ethanol by i.p. injection or gavage before burn, a ~47% increase (*P* < 0.05) above all other groups was observed. Taken together, the serum transaminase and LW : BW suggest that ethanol potentiates liver damage after burn injury irrespective of the route of intoxication.

### 3.6. I.P. or Gavage Administration of Ethanol Enhances Alveolar Wall Thickness after Burn

**Figure 8:**
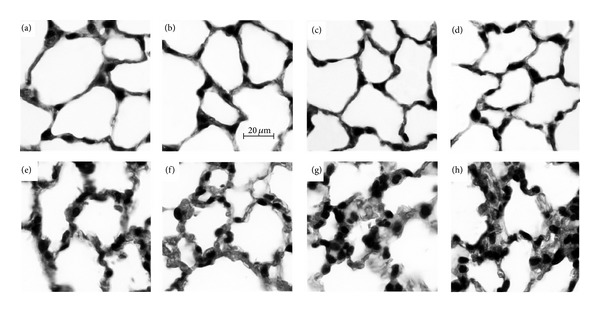
Histologic state of the lungs 24 hours after injury. Sham injured mice receiving i.p. (a) and gavage (b) control, or i.p. (c) and gavage (d) ethanol have normal appearing alveoli. Burn injury alone receiving i.p. (e) and gavage (f) control display an increase in alveolar wall thickness compared to sham injured animals (a–d). Intoxication by i.p. injection (g) or gavage (h) prior to burn results in further amplified alveolar wall thickness and cellularity.

**Figure 9:**
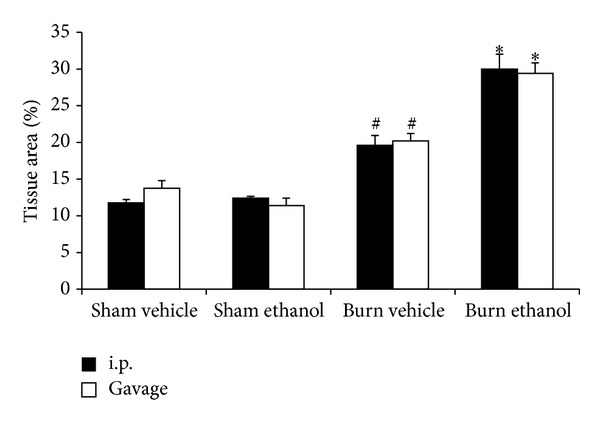
Quantification of pulmonary congestion 24 hours after injury. *P < 0.05 compared to Sham and Burn Vehicle groups. ^#^ *P* < 0.05 compared to Sham groups. Data are presented at mean values ± SEM. *N* =  4–6 animals per group.

At 24 hours after intoxication and burn injury, there is a marked increase in the thickness of the alveolar wall and increased cellularity, which is more pronounced than after burn alone (Figures 8 and 9). The alveolar wall thickness and cellularity was quantified using imaging software to measure the area of lung tissue in 10 high power fields per animal which is reported as a percentage of the entire field of view. A significant increase in tissue area, corresponding to a relative decrease in air space, was found after burn injury, compared to sham animals (*P* < 0.05). Intoxication increased the tissue area after burn regardless of how it was achieved (*P* < 0.05), indicating a greater level of pulmonary congestion.

### 3.7. Neutrophil Accumulation and Pulmonary KC Levels Are Amplified after I.P. or Gavage Intoxication and Burn

**Figure 10:**
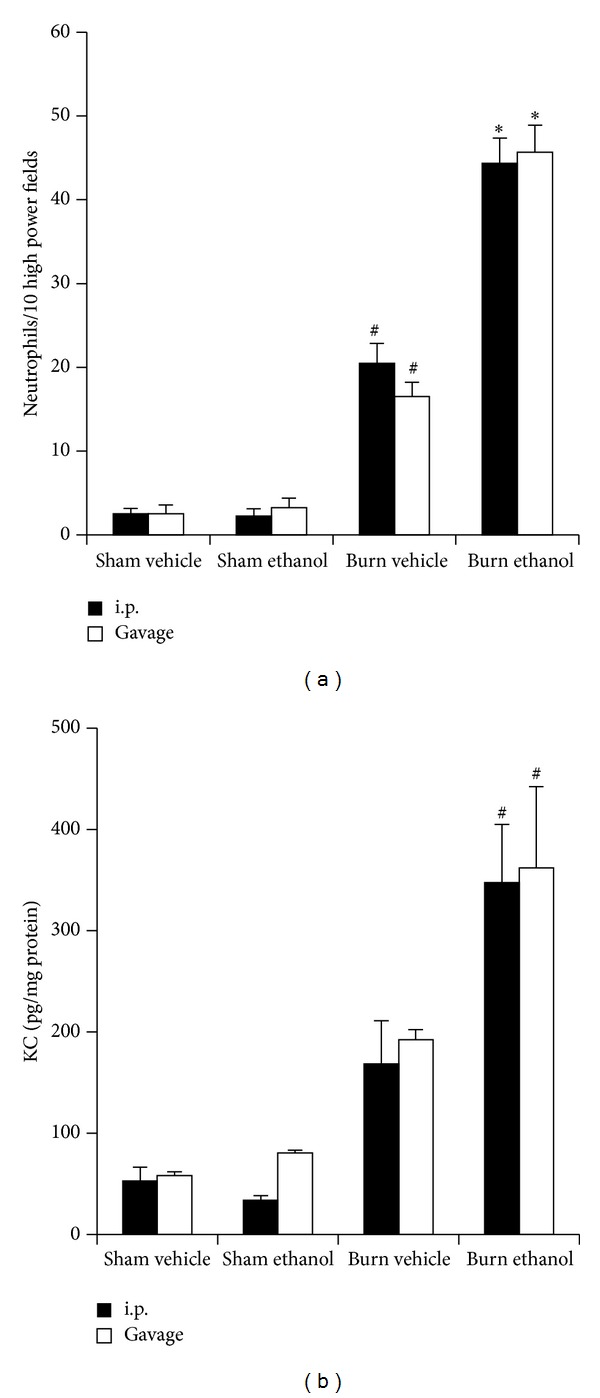
Pulmonary neutrophils in 10 high power (400x) fields of view (a) and pulmonary KC levels (b) 24 hours after injury. ^#^ *P* < 0.05 compared to Sham groups. *P < 0.05 compared to Burn Vehicle groups. Data are presented at mean values ± SEM. *N* =  4–8 animals per group.

Similar to previous studies [19, 20, 27], following the combined insult of i.p. ethanol injection and burn, there was a 20-fold increase in pulmonary neutrophils compared to sham animals (*P* < 0.05) and a 2-fold increase over burn alone (*P* < 0.05) (Figure 10(a)). This neutrophil accumulation in i.p. intoxicated animals corresponded to a 6-fold increase in KC compared to sham animals (*P* < 0.05) and a 2-fold elevation compared to burn alone (Figure 10(b)). When an equal amount of ethanol was given by gavage, similar results were observed with intoxicated burned mice having neutrophil numbers and KC levels that were 15- and 6-fold above sham animals (*P* < 0.05) and 2.5- and 2-fold above burn alone, respectively (Figure 10). No significant differences between i.p. and gavage mice within treatment groups were found suggesting that intoxication enhances post burn pulmonary neutrophil accumulation and KC regardless of administration route.

## 4. Discussion

The studies above indicate that in the context of burn injury in mice, intoxication at an equivalent BAC and duration exacerbates organ inflammation and damage to a similar extent whether given by oral gavage or i.p. injection. Equal BACs at the time of injury resulting in comparable amounts of organ damage are consistent with findings that suggest ethanol acts through worsening ischemic damage [28], altering cytokine networks [29, 30], and impairing immune responses [31] after burn injury and perhaps not through interactions at the site of absorption. The near infinite water solubility of ethanol allows for a quick distribution throughout the blood and we observed peak BACs near 30 minutes when ethanol was administered by oral gavage or i.p. injection. As seen in Figure 1, identical doses of ethanol by both paradigms resulted in nearly the same BAC at 30 minutes, 1 hour, and 4 hours after administration. The absorption time and BAC of the i.p. mice agree with our previously published studies [17, 26, 30] but the equivalency of BAC profiles between gavage and i.p. injection is in contrast with the work by Livy et al. [32] who concluded that in mice, ethanol given by gavage resulted in a lower BAC than an equivalent amount of ethanol given by i.p. injection. They proposed that this discrepancy may be due to metabolism by gastric alcohol dehydrogenase, which only occurs when ethanol traverses the gastrointestinal tract. While the reasons for the contrasting results are unclear, several possibilities, including differences in ethanol amount (3.8 g/kg versus 1.12 g/kg), the volume of ethanol administered (up to 0.41 mL versus 0.15 mL (i.p.)), and discrepancy between the vehicle used in i.p. injections (water versus saline) may have been contributing factors. Nevertheless, the mice used in our studies, which were given equal doses of ethanol by gavage and i.p. injection, demonstrated equivalent BAC profiles and an over exuberant inflammatory response after burn. A further discussion regarding considerations of i.p. and gavage ethanol administration can be found elsewhere as reviewed by D'Souza El-Guindy et al. [33].

A neutrophilic leukocytosis is seen in a variety of illnesses and conditions and is widely regarded an indicator of infection or inflammation. Trauma can also induce a leukocytosis where it is considered an acute phase marker and is clinically associated with increased morbidity and mortality risk [34]. We observed, in Figure 2, that intoxication by either paradigm induced a similar granulocytic leukocytosis at 24 hours after burn. The sequestration of these circulating neutrophils in end organs after injury is proposed as a major mechanism in the pathogenesis of multiple organ failure [35]. We and others have shown that intoxication at the time of burn leads to increased neutrophil infiltration into the gut, liver, and lungs of mice within 24 hours [8, 9, 36], Figure 10(a). Furthermore, prevention of neutrophil transmigration using ICAM knockout mice in this setting decreased pulmonary inflammation [20], highlighting the important role of neutrophil infiltration in this setting.

Circulating neutrophils migrate from the blood into tissues along a density gradient of chemoattractants, which in the mouse include KC. In mice, a burn injury increases pulmonary KC and ethanol has been shown to amplify this accumulation both in the absence [19–21] and presence of an intratracheal infection with *Pseudomonas aeruginosa* [30, 37]. We observed that both ethanol paradigms increase pulmonary KC equally after burn (Figure 10(b)) and this corresponded to increased neutrophil numbers in the lung (Figure 10(a)). The leukocytosis after intoxication and burn, together with an increase in neutrophil chemoattractants, likely plays a key role in the subsequent pulmonary inflammation and appears to be independent of the method of ethanol administration.

Elevated levels of circulating IL-6 also correlate with mortality risk in trauma patients [38] and are further increased when intoxication precedes burn injury [7, 17, 39]. We confirm our previous findings that burn alone increases serum IL-6 levels in mice and intoxication at the time of injury raises circulating IL-6 even further. We now report that this amplified IL-6 level when intoxication precedes burn injury is not affected by the route of ethanol administration (Figure 3). IL-6 in the setting of ethanol and burn has a causative role in intestinal damage [22] and pulmonary inflammation [21] though the source of systemic IL-6 is currently unknown. Of interest is the finding that the combination of intoxication and burn injury leads to greater bacterial translocation than either insult alone [26], which may incite a hepatic response, including IL-6 production.

Intestinal bacteria and lipopolysaccharide (LPS) that enter the portal system encounter Kupffer cells in the liver. This interaction between the intestinal microbiome and liver homeostasis is known as the “gut-liver axis” and plays a role in a myriad of diseases. Both burn and alcohol are known manipulators of the gut-liver axis and the combination of these insults has been shown to synergistically worsen hepatic damage in mice [18]. Clinically, liver function closely correlates to mortality risk after burn [40] and the importance of the gut-liver axis is highlighted by animal studies demonstrating improved outcomes after trauma when the gut is prophylactically sterilized with antibiotics [41, 42]. Increased liver damage and LPS stimulation may lead to hepatic production of excessive amounts of systemic IL-6, which as mentioned above, plays a causative role in the increased pulmonary inflammation of burned intoxicated mice. This is of clinical significance because multiple organ failure is common after a substantial injury and the lungs are among the first organs to fail.

We now report gavage or i.p. intoxication potentiated postburn intestinal damage as demonstrated by histology (Figure 4) and villus length (Figure 5(a)). Furthermore, intestinal damage corresponded to an increase in bacterial translocation (Figure 5(b)) and hepatic damage as assessed by serum transaminase levels (Figure 6) and hepatic weight (Figure 7). These findings were independent of the ethanol administration route in our model and support the idea of an altered gut-liver axis when intoxication is present at the time of burn injury. Of note is the rise in serum IL-6 levels (Figure 3) that mimic the pattern of damage observed in the liver (Figure 6).

Increased serum IL-6 is linked to poor survival in patients with acute respiratory distress syndrome (ARDS) [43]. ARDS is characterized by inflammation and edema in the lung parenchyma leading to impaired gas exchange. When examined by histology (Figure 8), the lungs of mice from both gavage and i.p. paradigms appear congested relative to all other treatment groups. When the amount of tissue relative to air space was quantified (Figure 9), the alveolar wall thickening and increased cellularity seen visually was found to be increased after burn and further increased with prior intoxication. This finding agrees with our previously reported work with i.p. injected mice [21] and is unaffected by the route of administration.

## 5. Conclusions

The socioeconomic impact and clinical relevance of intoxication at the time of burn injury merit in-depth investigation into the mechanisms for worsened outcome in these patients. Animal studies using mice offer controlled conditions, manipulatable genomes, and pharmacologic interventions not available in humans. An important variable is the level of intoxication achieved before burn and while historically animal studies have administered known amounts of ethanol by i.p. injection, oral gavage is considered a more physiologic method of intoxication. We now describe that postburn inflammation and damage in the ileum, liver, and lungs of mice are exacerbated to an equal extent when preceded by intoxication achieved by i.p. injection or gavage. Furthermore, the administration route had no impact on the hematologic changes observed when intoxication precedes burn. Taken together our data suggest that either i.p. injection or gavage is appropriate for studying the effects of ethanol on postburn inflammation and response.
